# Therapeutic effect of gold nanoparticles on DSS-induced ulcerative colitis in mice with reference to interleukin-17 expression

**DOI:** 10.1038/s41598-019-46671-1

**Published:** 2019-07-15

**Authors:** Amira M. Abdelmegid, Fadia K. Abdo, Fayza E. Ahmed, Asmaa A. A. Kattaia

**Affiliations:** Department of Histology and Cell Biology, Faculty of Medicine, Zagazig University, Zagazig, Egypt

**Keywords:** Ulcerative colitis, Gastrointestinal models, Experimental models of disease

## Abstract

Ulcerative colitis (UC) is among the most challenging human diseases. Nanotechnology has incontestable promising outcomes in inflammatory bowel diseases. This study aimed to investigate the therapeutic effect of naked gold nanoparticles (AuNPs) on dextran sodium sulphate (DSS) induced ulcerative colitis in mice. We also examined the expression of interleukin-17 (IL-17) following AuNPs treatment. Mice were randomly divided into control, DSS and DSS+ AuNPs groups. Severity of colitis was assessed by disease activity index (DAI) measurement. At the end of the experiment, the final body weights were recorded. The colon was dissected and processed for histopathological examinations by light and electron microscopes. Colon homogenates were prepared for assay of tissue malondialdehyde (MDA) and real-time PCR analysis of IL-17A. Immunohistochemical localization of IL-17A was carried out. Scanning electron microscopy (SEM) and Energy Dispersive X-ray (EDX) detector were used to detect the presence of AuNPs in the colonic tissue of DSS+ AuNPs groups. Our results showed that AuNPs effectively targeted the colonic tissue, and reduced changes induced by DSS. The underlying mechanisms could be related to anti-oxidant effect (as evident by decreasing tissue MDA) and anti-inflammatory potential of AuNPs. Our study draws attention to as a novel therapeutic strategy for treating UC.

## Introduction

Inflammatory bowel disease (IBD) is a complex, multifactorial, immune mediated gastrointestinal disorder characterized by chronic relapsing inflammation in the gut. IBD involves ulcerative colitis (UC) and Crohn’s disease (CD)^1^. UC is characterized clinically by diarrhea and rectal bleeding which is usually mixed with mucous pus. The diagnosis of UC is based on a combination of clinical, endoscopic, and histologic features^2^. The life quality of patients with ulcerative colitis can be limited by complications such as bowel perforation, toxic mega colon, surgical complications and increased risk of colorectal cancers^3^.

Treatment of IBD aims to block the pathogenic steps of the inflammatory cascade^4^. Interleukin-17 (IL-17) has also gained increasing attention as a key mediator in the pathogenesis of intestinal inflammation^5,6^. It acts as a potent inflammatory interleukin that activates the expression of other pro-inflammatory cytokines^7^. IL-17 is produced mainly by T helper 17 cells (TH17 cells) and other sources including natural killer cells, mast cells and neutrophils^8^. IL-17 cytokine family comprises IL-17A, IL-17B, IL-17C, IL-17D, IL-17E (IL-25) and IL-17F^9^. IL-17 level is up-regulated in inflamed mucosa from IBD patients^10^. The disease severity is correlated with the IL-17 level in peripheral blood mononuclear cells from UC patients^11^.

Several regimens have been used in the treatment of IBD. However, their efficacy continues to be unsatisfying. Side effects of systemic therapies and poor targeting of oral drugs represent frequent therapeutic challenges. Also, complications of drugs encourage for alternative therapy for UC^4,12^. Nanotechnology is an emerging technology in the medical area with applications in the treatment and prevention of chronic diseases^13^. Therapeutic nanoparticles could provide amazing advantage in the treatment of IBD^14^. Gold nanostructures have been extensively investigated due to their versatility. The unique optical and photo thermal properties, biocompatibility, non-immunogenicity, antimicrobial properties and facile surface chemistry are some of valuable features of AuNPs^15^. AuNPs were applied as a therapeutic modality in different diseases such as rheumatoid arthritis, diabetes mellitus and chronic myeloid leukemia^16^.

Based on this background, this study aimed to investigate the therapeutic effect of naked AuNPs on DSS induced ulcerative colitis in mice. We also examined the expression of IL-17 following AuNPs treatment.

## Results

### AuNPs characterization

**Figure 1:**
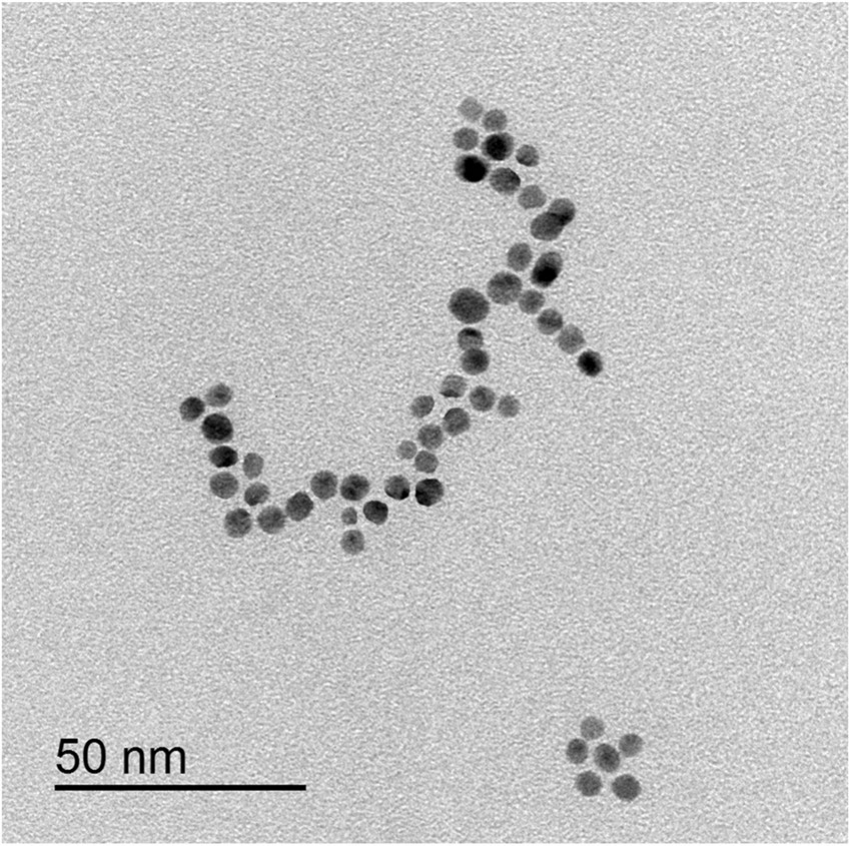
Transmission electron microscope image showing average diameters of AuNPs.

The AuNPs used in this study had an average diameter of 5 nm, as revealed by transmission electron microscopy (Fig. 1).

The control subgroups Ia and Ib showed nearly similar results Consequently, only results of the control subgroup Ia were presented in tables and figures. Results of the control subgroup Ib were listed at the end of this section.

### Body weight measurements

Statistical analysis of the final body weight measurements revealed a significant decrease in the DSS group (0.8 fold) compared to the control (*p* < 0.05). There was a non-statistical significant difference in the DSS+ AuNPs group compared to the control (Table 1).

**Table 1:** Body weights, colon length and tissue malondialdehyde (MDA) level in different groups.

	Control group	DSS group	DSS+ AuNPs group
Body weight (g), 14^th^ day	26.45 ± 0.29	24.13 ± 0.36^∗∗a^	26.1 ± 0.36^∗b^
Body weight (g), by the end of experiment	28.67 ± 1.8	22.7 ± 0.26^∗a^	27.03 ± 0.4^∗b^
Colon length (cm)	8.9 ± 0.26	7.01 ± 0.23^∗∗a^	8.46 ± 0.24^∗b^
MDA (nmol/g protein)	1.233 ± 0.09	5.45 ± 0.31^∗∗a^	2.067 ± 0.15^∗∗b^

Values are expressed as mean ± standard error of means (SEM) of n = 8 animals. ^a^*P* compared with control group. ^b^*P* compared with DSS group. ^∗^Significant difference (*P* < 0.05). ^∗∗^Highly significant difference (*P* < 0.001).

### Colon length measurements

The length of the colon was significantly shorter in the DSS group compared with the control group (*p* < 0.001), whereas the DSS+ AuNPs group was comparable to the control group (Table 1).

### Disease activity index (DAI) measurement

**Figure 2:**
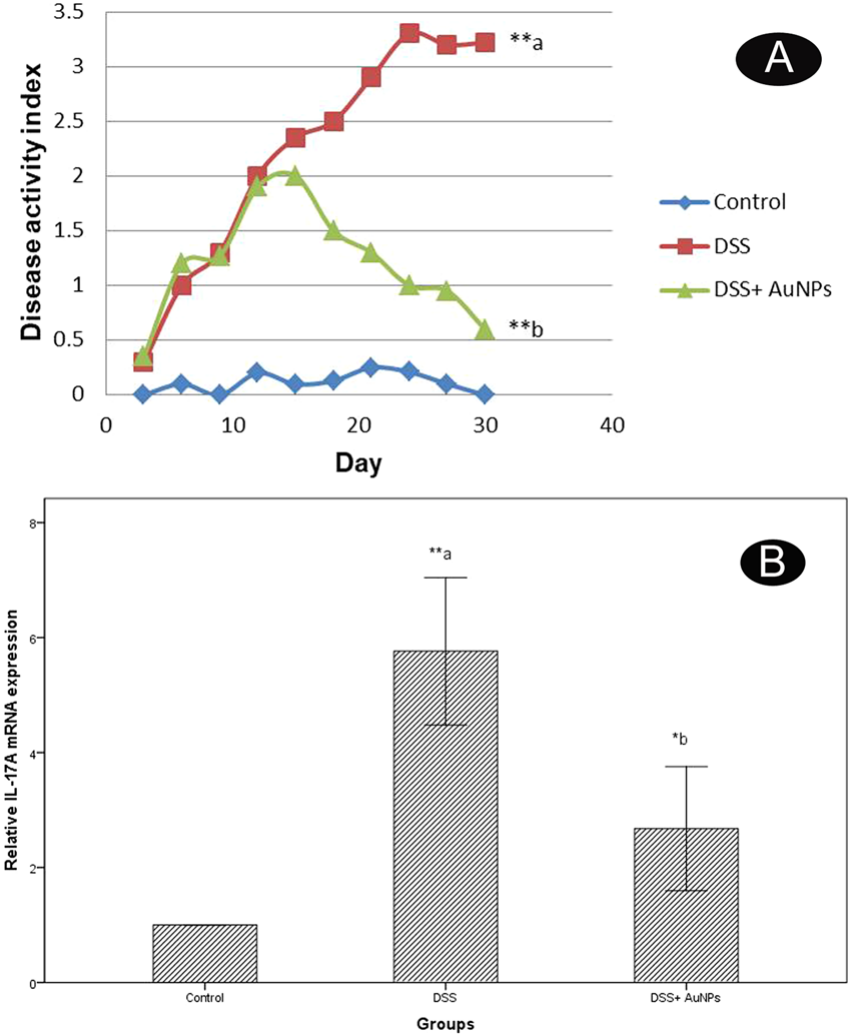
(**A**) Changes in the disease activity index (DAI). a: *P* compared with control group; b: *P* compared with DSS group; Asterisks * and ** denote *p* < 0.05 and *p* < 0.001 respectively; n = 8. (**B**) Real-time PCR analysis of IL-17A expression levels in the colons of study groups. Values are estimated as a fold-increase compared to IL-17A expression in the calibrator control which is equal to 1. a: *P* compared with control group; b: *P* compared with DSS group; Asterisks * and ** denote *p* < 0.05 and *p* < 0.001 respectively; n = 8.

DAI showed a highly significant increase in the DSS group compared to the control (*p* < 0.001). On the other hand, the DSS+ AuNPs group revealed a highly significant decrease compared to DSS group (Fig. 2).

### Biochemical results

- Tissue malondialdehyde (MDA) levels
  Measurements of MDA levels revealed a highly significant increase in the DSS group (4.4 folds) compared to control group as (*p* < 0.001). There was a non-statistical significant difference in the DSS+ AuNPs group treated group compared to the control group (Table 1)
- IL-17A gene expression results
  There was a highly significant increase (5.8 folds) in IL-17A gene expression in the DSS group (*p* < 0.001) when compared to normal controls. On contrary, There was a significant decrease in the DSS+ AuNPs group when compared to the DSS group (*p* < 0.05) (Fig. 2B).

### Histopathological results

- Light microscope results

#### Results of Haematoxylin & Eosin (H&E) stain

**Figure 3:**
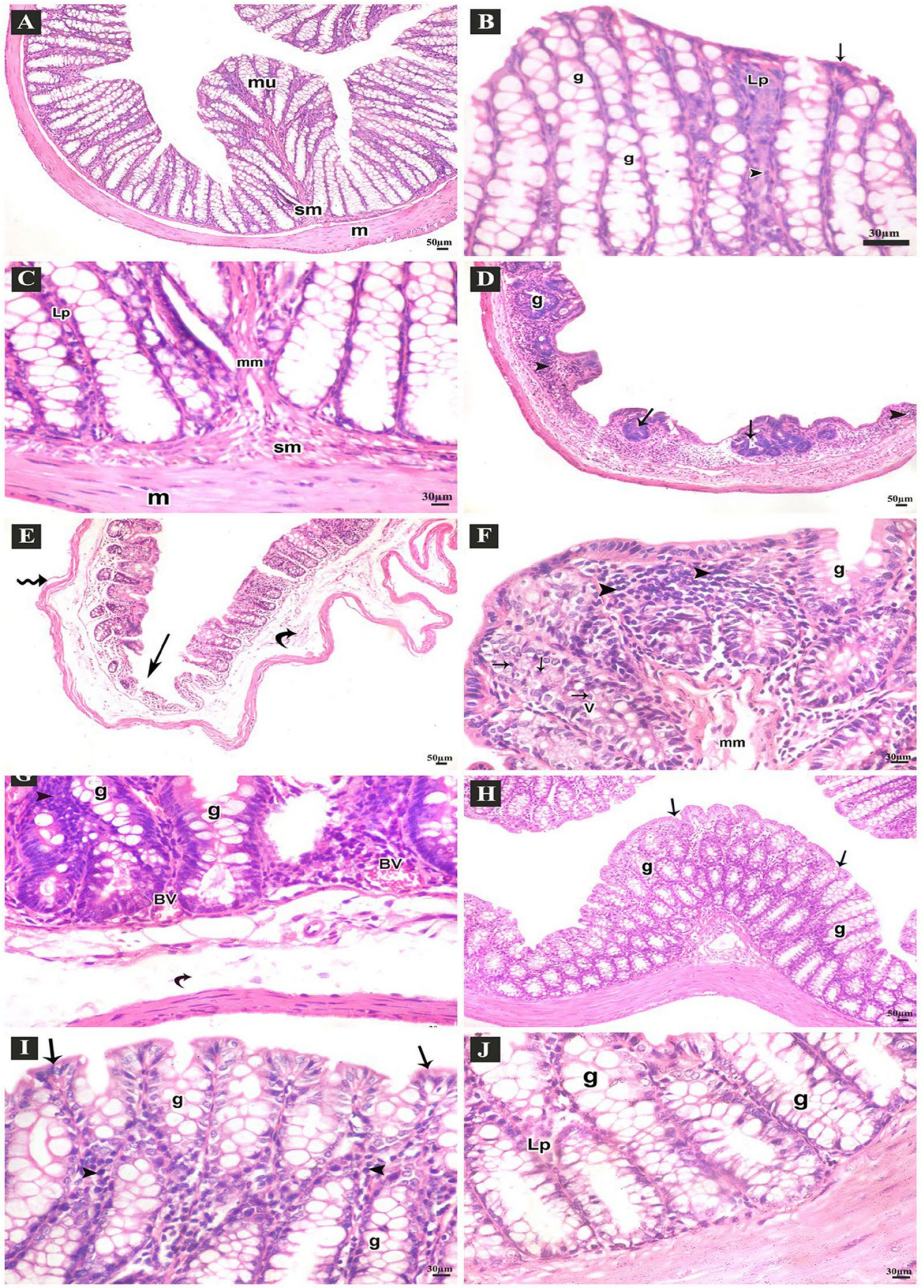
H&E stained-sections of the colon of adult male mice. (**A**–**C**: Control group **A**) Normal arrangement of the lining layers; mucosa (mu), submucosa (sm) and musculosa (m) (100x). (**B**) Apical parts of the mucosa; absorptive columnar cells (arrows), numerous goblet cells (g), thin lamina propria (Lp), few lymphocytes (arrow heads) (400x). (**C**) Basal parts of the mucosa; thin lamina propria (Lp), normal muscularis mucosa (mm), submucosa (sm) and musculosa (m) (400x). (**D**–**G**: DSS group **D**) Few abnormal crypts (arrows), scanty goblet cells (g), diffuse inflammatory cell infiltrations (arrow heads) (100x). (**E**) Ulcerated mucosa (arrows), submucosal widening (curved arrows), separated muscle fibers of musculosa (zigzag arrows) (100x). (**F)** Apical parts of the mucosa; few goblet cells (g), many vacuolated columnar cells (V), some cells with small dark nuclei (arrows), intense inflammatory cell infiltrations (arrow heads), separated muscle fibers of muscularis mucosa (mm) (400x). (**G**) Basal parts of mucosa; goblet cells (**G**), congested blood vessels (BV), submucosal edema (curved arrows), many inflammatory cell infiltrations (arrow heads) (400x). (**H**–**J**: DSS+ AuNPs group H) Preserved crypts (arrows), many goblet cells (g) (100x). (**I**) Apical parts of the mucosa; columnar cells (arrows) and many goblet cells (g), thin lamina propria (Lp), some inflammatory cells (arrow heads) (400x). (**J**) Basal parts of the crypts; many goblet cells (g), thin lamina propria (Lp) (400x).

Examination of the H&E stained sections of the colon of the control group revealed normal arrangement of the lining layers: mucosa, submucosa and musculosa. The crypts were lined by columnar absorptive cells with acidophilic cytoplasm and basal oval nuclei. Numerous goblet cells appeared with basal nuclei and vacuolated cytoplasm. There was thin lamina propria containing few lymphocytes. Prominent muscularis mucosa, normal submucosa and musculosa were seen (Fig. 3A–C). Sections of DSS group showed disturbed architecture of colon. There were few crypts, intense inflammatory cell infiltrations, mucosal ulcers and submucosal edema, separated muscle fibers of musculosa. The crypts were lined with few goblet cells, many vacuolated cells and some cells with dark nuclei. Congested blood vessels were also observed (Fig. 3D–G). Sections from DSS+ AuNPs group showed apparently normal structure of the lining layers of the colon. Preserved crypts with many goblet cells were seen. Some inflammatory cell infiltrations in the lamina propria were still observed (Fig. 3H–J).

**Figure 4:**
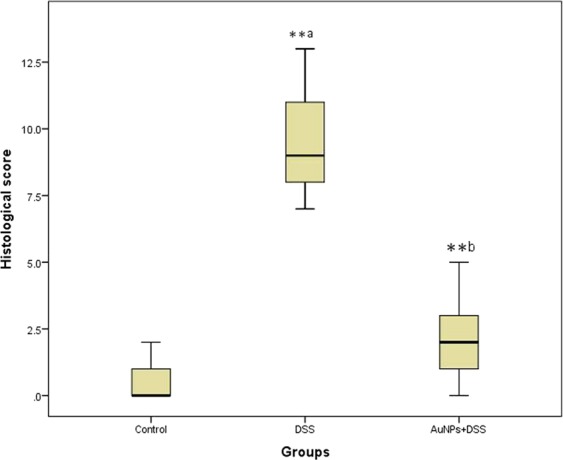
Histological damage scores by groups. The boxplot shows the median value (solid horizontal line), the interquartile range (box outline), and the minimum and maximum values (whiskers); a: *P* compared with control group; b: *P* compared with DSS group; ∗: significant difference (*P* < 0.05); ∗∗: highly significant difference (*P* < 0.001).

We summarized the histological changes using a scoring system that weighted the severity of inflammatory cell infiltrate, ulceration, crypt damage and edema. The total damage score were represented in (Fig. 4).

#### Results of toluidine blue stain

**Figure 5:**
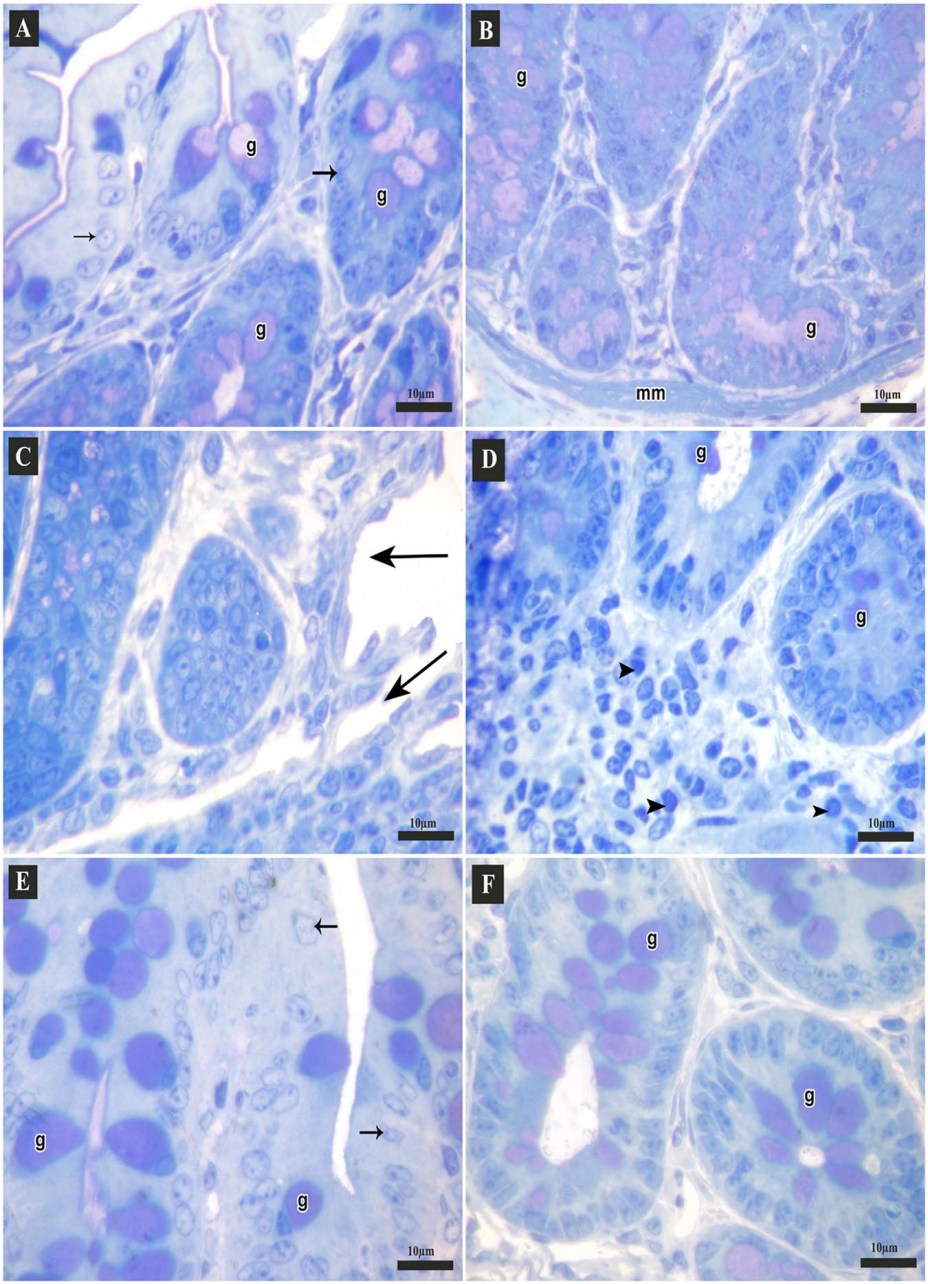
Semi-thin sections of the colon of adult male mice (Toluidine blue stain, 1000x). (**A,B**: Control group **A**) Apical parts of the mucosa; columnar cells (arrows) and many goblet cells (g) that may open into the lumen. (**B**) Basal parts of the crypts lined with many goblet cells (g), muscularis mucosa (mm). (**C**,**D**: DSS group **C**) Apical parts of mucosa with mucosal ulcers (arrows). (**D**) Basal parts of the crypts; few goblet cells (g), diffuse inflammatory cell infiltrations (arrow heads). (**E,F**: DSS+ AuNPs group **E**) Apical parts of the mucosa, columnar cells (arrows), many goblet cells (g). (**F**) Basal parts of the crypts, many goblet cells (g).

Toluidine blue-stained semi-thin sections of the control group showed parts of crypts lined with columnar cells and many goblet cells. Some of them opened into the lumen, while in the basal part muscularis mucosa was seen (Fig. 5A,B). DSS group showed mucosal ulcers. The crypts were lined with few goblet cells. Diffuse inflammatory cell infiltrations were noticed in lamina propria (Fig. 5C,D). Section from DSS+ AuNPs group revealed nearly normal colonic crypts that lined with columnar cells and many goblet cells (Fig. 5E,F).

#### Results of Mallory’s trichrome stain

**Figure 6:**
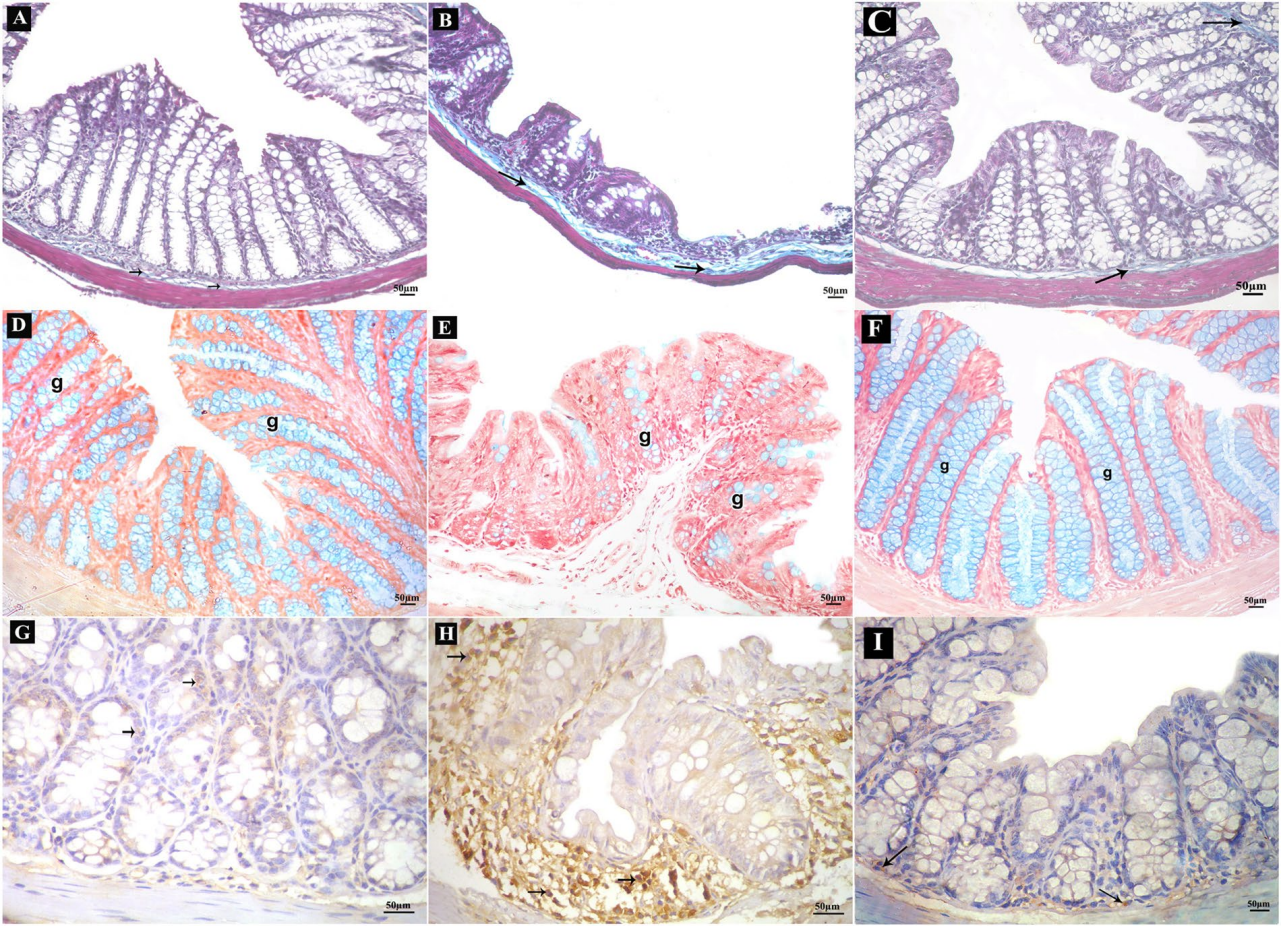
Sections of the colon of adult male mice. (**A**–**C)** Mallory’s trichrome-stained sections (200x); collagen fibers (arrows). (**A**) Control group showing few collagen fibers, (**B**) DSS group showing numerous collagen fibers, (**C**) DSS+ AuNPs group showing some collagen fibers. (**D**–**F**) Alcian blue-stained sections (200x); goblet cells (g). (**D**) Control group with abundant goblet cells, (**E**) DSS group with few goblet cells, (**F**) DSS+ AuNPs group with many goblet cells. (**G**–**I**) Immune histochemical-stained sections for IL-17A (400x); brown positive cytoplasmic reactions (arrows). (**G**) Control group showing reactions in few cells, (**H**) DSS group showing reactions in many cells, (**I**): DSS+ AuNPs group showing reactions in some cells.

Examination of Mallory’s trichrome-stained sections of the control group showed few collagen fibers in lamina propria and submucosa (Fig. 6A). Sections from DSS group showed numerous collagen fibers in the lamina propria and submucosa (Fig. 6B). DSS+ AuNPs group revealed some collagen fibers in lamina propria and submucosa (Fig. 6C).

#### Results of alcian blue stain

Alcian blue-stained sections of the control showed mucosal crypts lined by abundant goblet cells (Fig. 6D). DSS group showed crypts lined by few goblet cells (Fig. 6E). DSS+ AuNPs group revealed that the crypts were lined by many goblet cells (Fig. 6F).

#### Immune histochemical results

Immune histochemical-stained sections for IL-17A of the control group showed brown positive cytoplasmic reactions in few cells (Fig. 6G). DSS group revealed positive immune reactions in many cells (Fig. 6H). DSS+ AuNPs group showed positive immune reactions in some cells (Fig. 6I).

- Electron microscope results

**Figure 7:**
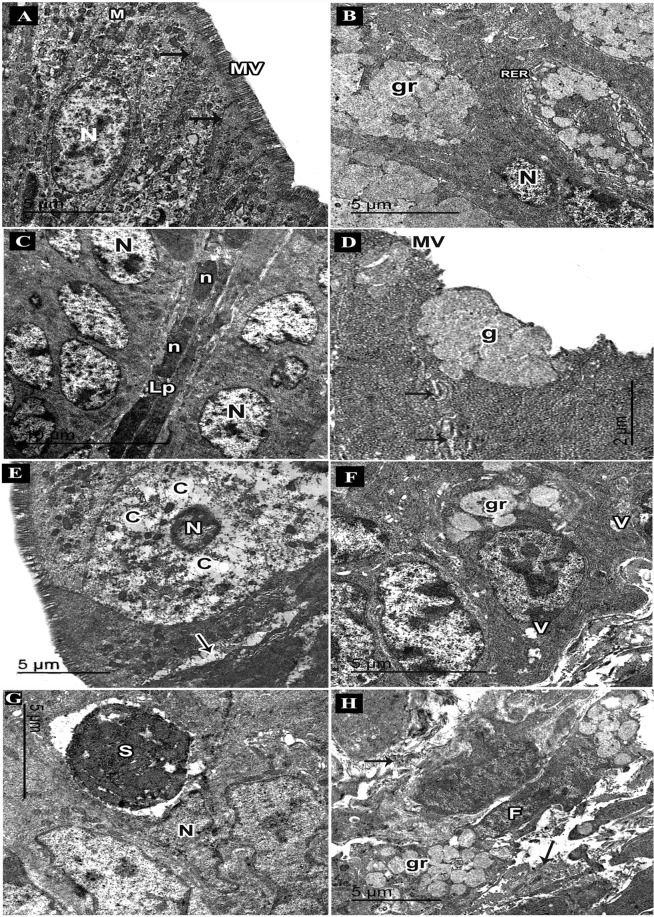
An electron micrograph of ultrathin sections in the colon of adult male mice. (**A**–**C**: Control group. **A**) Columnar cell nucleus (N), microvilli (MV), mitochondria (M), cell junctions (arrows). (**B**) Goblet cells nuclei (N), rough endoplasmic reticulum (RER), mucous granules (gr). (**C**) Lamina propria (Lp), fibroblasts with elongated nuclei (n), columnar cells nuclei (N). (**D–I**: DSS group. **D**) Columnar cells with few microvilli (MV), abnormal cell junctions (arrows), goblet cell (g). (**E**) A degenerated cell with dark small nucleus (N); rarified cytoplasm (**C**), areas of separations between cells (arrows). (**F**) Abnormal goblet cell with few mucous granules (gr), vacuoles (V). (**G**) Atypical goblet cell with an electron dense condensed secretion (S) surrounded by a space, nucleus (N). (**I**) Lamina propria with abundant collagen fibres (arrows), fibroblasts (F) with secretory granules (gr).

Electron microscope examination of the ultrathin sections of the control colon showed absorptive columnar cells with regular oval euchromatic nuclei, numerous microvilli and plentiful mitochondria. Well-developed cell junctions were seen (Fig. 7A). Numerous goblet cells appeared with basal euchromatic regular nuclei, plentiful rough endoplasmic reticulum and several apical mucous granules that might coalesce (Fig. 7B). A thin core of lamina propria containing fibroblasts with elongated nuclei was noticed (Fig. 7C).

**Figure 8:**
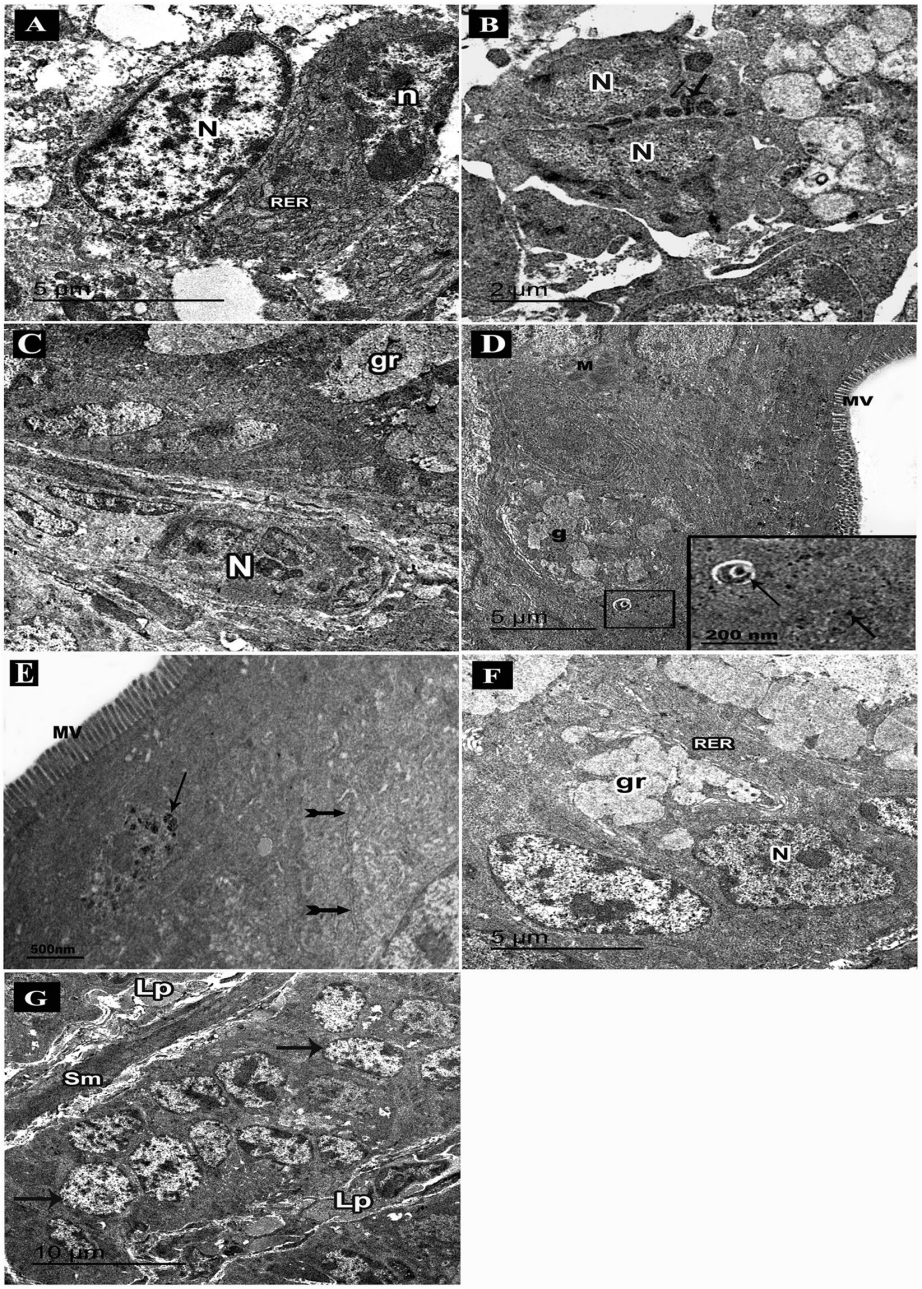
An electron micrograph of ultrathin sections in the colon of adult male mice. (**A–D**: DSS group. **A**) Intraepithelial infiltrating plasma cell with cartwheel-shaped nucleus (n), rough endoplasmic reticulum (RER), nucleus of columnar cell (N). (**B**) An eosinophil with bilobed nucleus (N) and specific granules (arrows). (**C**) Infiltrating neutrophil with segmented nucleus (N), a goblet cell with mucous granules (gr). (**D**–**G**: DSS+ AuNPs group. **D**) Columnar cells with abundant microvilli (MV) and mitochondria (M), a part of goblet cell (g). An inset: magnification of the boxed part showing electron dense AuNPs (arrows) in the cytoplasm and within a lysosome (**E**) Columnar cells with abundant microvilli (MV), normal cell junction (bifid arrows), a lysosome containing electron dense AuNPs (arrows). (**F**) Goblet cells with mucous granules (gr), abundant rough endoplasmic reticulum (RER) and basal nuclei (N). (**G)** Columnar cells (arrows), a thin core of lamina propria (Lp), smooth muscle cells of muscularis mucosa (sm).

Ultrathin sections of the colon from the DSS group showed columnar cells with few microvilli and abnormal cell junctions (Fig. 7D). Damaged cells appeared with dark small nuclei, rarified cytoplasm and degenerated organelles. Separations between cells could be observed (Fig. 7E). Abnormal goblet cells had few apical mucous granules. Some vacuoles were also seen (Fig. 7F). Atypical goblet cells appeared with electron dense condensed secretions surrounded by a space (Fig. 7G). Parts of lamina propria showed abundant collagen fibers and fibroblasts with secretory granules containing immature collagen (Fig. 7H). Intra epithelial infiltrating plasma cells with characteristic cartwheel nuclei and extensive array of rough endoplasmic reticulum were seen (Fig. 8A). Infiltrating eosinophils appeared with bilobed nuclei and specific granules having an electron dense crystalline core surrounded by a less dense material (Fig. 8B). Infiltrating neutrophils appeared in the lamina propria with their segmented nuclei (Fig. 8C).

Ultrathin sections of DSS+ AuNPs treated group showed luminal surface of columnar cells with numerous microvilli, mitochondria and normal cell junction. Goblet cells with many mucous granules, abundant rough endoplasmic reticulum and basal nuclei were observed (Fig. 8D–F). AuNPs were demonstrated in the cytoplasm and some lysosomes (Fig. 8D,E). There was relatively thin core of lamina propria containing connective tissue cells (Fig. 8G).

### Scanning electron microscopy (SEM) and Energy Dispersive X-ray (EDX) detector results

**Figure 9:**
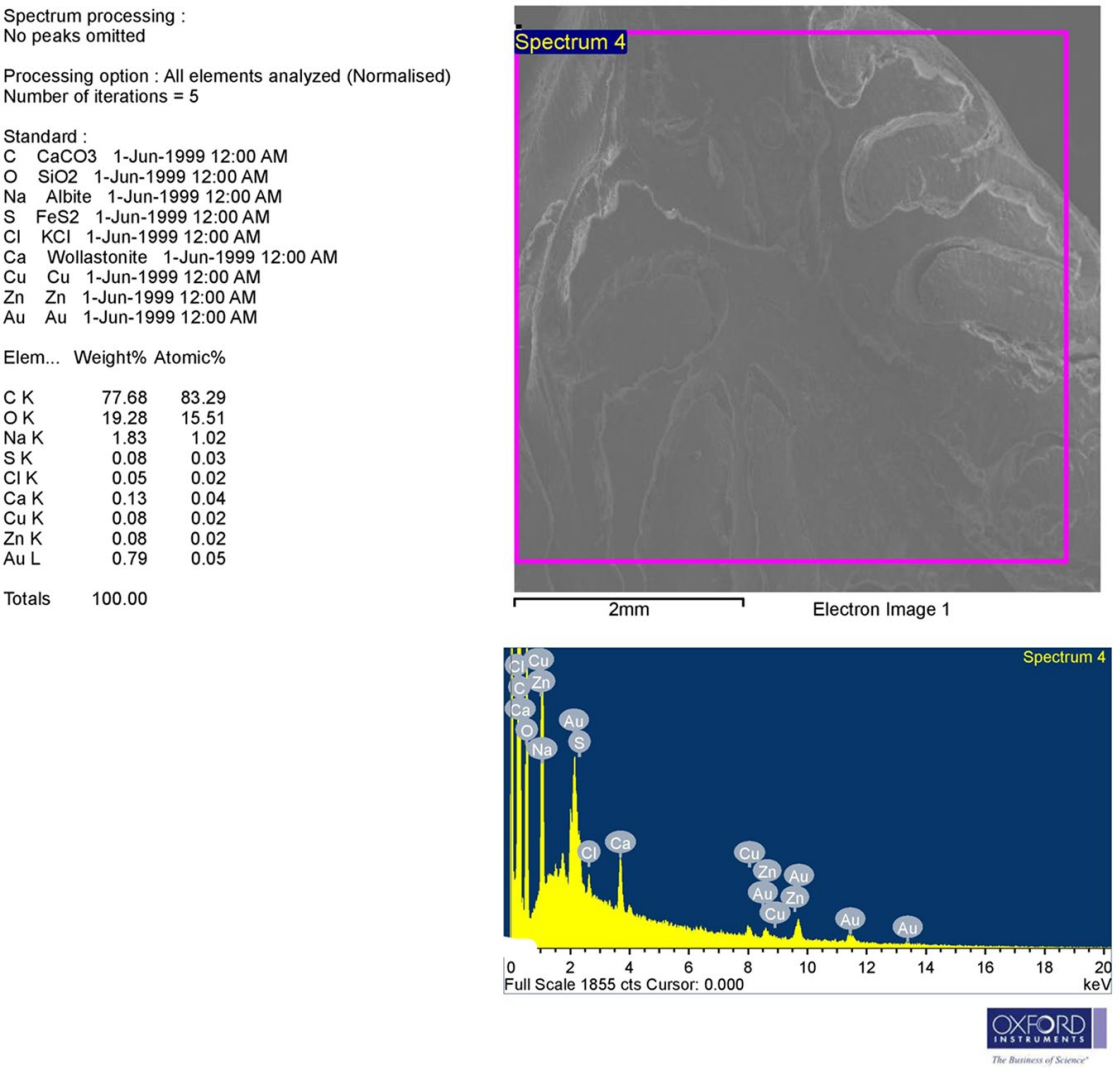
The upper part: SEM image; backscattered electron image displays compositional contrast which results from different elements. The lower part: EDX analysis allows identifying these elements and their relative proportion (atomic % and weight %) by the generation of an x-ray spectrum from the entire scan area of the SEM. The Y-axis depicts the counts of x-rays and the X-axis shows their energy level (KeV). The position of the peaks identifies the elements, while the peaks height helps in the quantification of each element. Here, EDX spectrum showing significant amounts of Au (one prominent peak at 2.2 KeV with other weak peaks).

Analysis of SEM image and EDX spectrum revealed the presence of significant amounts of AuNPs (Fig. 9).

### Morphometric results

Statistically analyzed results for mucosal and submucosal thickness measurements were represented in (Table 2). The number of goblet cells, the area percentage of collagen fibers and the area percentage of positive anti-IL-17A immune reactions were summarized in (Table 3).

**Table 2:** Mucosal and submucosal thickness of different groups.

	Control group	DSS group	DSS+ AuNPs group
Mucosal thickness (µm)	363.3 ± 13.58	151.3 ± 14.93^∗∗a^	344.79 ± 14.84^∗∗b^
Submucosal thickness (µm)	38.44 ± 1.9	121.17 ± 11.53^∗∗a^	40.21 ± 3.03^∗∗b^

Values are expressed as mean ± standard error of means (SEM) of n = 8 animals. ^a^*P* compared with control group. ^b^*P* compared with DSS group. ^∗^Significant difference (*P* < 0.05). ^∗∗^Highly significant difference (*P* < 0.001).

**Table 3:** Number of goblet cells, area percentage of collagen fibers and area percentage of positive anti- IL-17A immune reactions in different groups.

	Control group	DSS group	DSS+ AuNPs group
Goblet cells	420.67 ± 41.87	121.17 ± 11.53^∗∗a^	290 ± 33.56^∗∗b^
Collagen fibers (%)	10.77 ± 0.15	26.48 ± 0.93^∗∗a^	12.50 ± 0.22^∗b^
Anti-IL-17A (%)	4.43 ± 0.33	36.17 ± 1.14^∗∗a^	7.47 ± 0.75^∗∗b^

Values are expressed as mean ± standard error of means (SEM) of n = 8 animals. ^a^*P* compared with control group. ^b^*P* compared with DSS group. ^∗^Significant difference (*P* < 0.05). ^∗∗^Highly significant difference (*P* < 0.001).

### Results for control subgroup Ib

Regarding body weight measurements (on day 14^th^ 26.09 ± 0.32 g, by the end of experiment 27.93 ± 0.23 g), colon length 8.38 ± 0.23 cm, DAI 0.16 ± 0.05, biochemical assays (MDA 1.07 ± 0.15 nmol/g protein, IL17 mRNA 1.02 ± 0.08) and histopathological results (mucosal thickness 351.3 ± 6.9 µm, submucosal thickness 39.02 ± 1.67 µm, histological score 0.3 ± 0.12, number of goblet cells 407.7 ± 7.46, area percentage of collagen fibers 9.24 ± 1.37 and area percentage of positive anti- IL-17A immune reactions 3.24 ± 0.53). There were non-statistical significant differences between control subgroup Ia and subgroup Ib.

## Discussion

Over the previous few decades, the prevalence and incidence of inflammatory bowel disease (IBD) have been developing international^17^. IBD is the fundamental risk factor for the development of gastrointestinal cancers, including colorectal cancer^18^.

In the present work, there was a significant loss in the body weights and a highly significant increase in the disease activity index (DAI) in the DSS group compared to the control group. Weight loss is an indicator of the severity of intestinal inflammation and correlates with the histopathological changes of colitis^19^. Weight loss could be contributed to the inflammatory state in IBD which results in mal-absorption of nutrients; a generalized catabolic state; and alterations in the levels of metabolic hormones affecting satiety^20,21^.

MDA is a marker for oxidative stress. MDA results from lipid peroxidation of polyunsaturated fatty acids and plays an important role in the tissue damage of the UC model^22,23^. In the present work, MDA levels were significantly elevated in the DSS group. DSS directly destructs the epithelial cells and triggers abnormal intrusion of microbial flora into the cell, which is capable of starting innate immune responses and leads to the generation of reactive oxygen species (ROS) and reactive nitrogen metabolites^24^. Abundant infiltrations by neutrophils are the main source of those harmful metabolites^25^.

In the current work, microscopic examination of the colon the DSS group revealed disrupted architecture as demonstrated by the high histological damage scores. Our results were in line with other investigators^26,27^. The crypts fail to regenerate as a result of colitis^28^. This might explain the presence of few damaged crypts in our study. Further, widened submucosa might be explained by the increased collagen deposition, edema or inflammatory cell infiltrations.

The augmented formation ROS plays an important role in the pathophysiology of UC. It initiates damage of cellular DNA, proteins, lipids and organelles, with disturbance of cell membrane integrity^29^. Other than, T regulatory (Treg) cells maintain immune cell homeostasis by suppressing the functions of other immune cell types, particularly T helper 17 (Th17) cells^30^. Th17/Treg imbalance may play a crucial role in this inflammatory process of UC^31^. Serum concentrations of Th17-related pro-inflammatory interleukins (IL-6, IL-17A, and IL-17F) will increase and Treg-related anti-inflammatory cytokines (IL-10, IL-2, and TGF-β) will decrease accordingly^32^. In the same context, we revealed that IL-17 gene expressions and IL-17 immune reactions were significantly up regulated in the DSS group compared with the control.

In UC-associated inflammatory processes, electron microscope examination of the DSS group revealed inflammatory cell infiltrations including eosinophils, neutrophils and plasma cells, which might represent an abnormal mucosal immune response to bacterial antigens. Recent data showed that eosinophils have key functions in IBD as well^33^. The inflamed colon contains multiple ligands for CD300f-expressing cells including monocytes, neutrophils and eosinophils which are recruited to the colon following DSS treatment^34^. IL-17 is also involved in the proliferation and chemotaxis of neutrophils^35^. Regarding goblet cells, morphometric analysis of alcian blue-stained sections showed a highly significant reduction in the number of goblet cells compared with the control group. These results were in agreement with others^36^. Impaired differentiation of goblet cells from intestinal stem cells was found in inflammatory bowel diseases^37^. Our ultra-structural examination revealed that goblet cells exhibited few mucous granules. Some cells appeared with abnormal condensed secretion. Secretion of abnormal mucins with decreased glycosylation and sulfation is associated with decreased viscosity and increased susceptibility to erosion and colonic inflammation^38,39^.

Organogold compounds have been used since the 1930s to treat rheumatic diseases. Gold compounds decrease the expression of inflammatory cytokines (IL-1, IL-6 and TNF) in rheumatoid arthritis patients^40^. Gold also inhibits the expression of nuclear factor kappa beta (NF-kB) which has been associated with chronic inflammatory diseases e.g. IBD^41^.

The role of nano-medicine in the gastrointestinal tract is regarded a promising therapeutic tool. Different types of nanoparticles have prospective uses in gastroenterology as they overcome the traditional treatment in several disorders^42^. AuNPs have begun to be actively used for diagnostic and therapeutic applications in several fields of nano-medicine^43^. AuNPs are attractive for biological applications due to their low toxicity, excellent biocompatibility, and shape-related optoelectronic properties^44^.

The studies of oxidative stress and inflammatory pathways have improved the understanding of the pathogenesis of UC. Hence, these pathways are the therapeutic targets in the new drug studies of UC^22,45^. In the current work, we used AuNPs of 5 nm size. The size of NPs is a critical parameter in the mechanism by which AuNPs inflammation. Sumbayev *et al*. proved that 5 nm AuNPs completely block the inflammatory process while 15 nm and 35 nm are less effective^46^. AuNPs (coated with tiopronin) smaller than 10 nm have shown interesting preferences over NPs larger than 10 nm in the terms of their capacity to interact with cells, they highly accumulated in cancer cells and distributed throughout the cytoplasm and nucleus^47^. Further, the smaller AuNPs are more quickly absorbed by the intestinal epithelial cells and more rapidly spread through the interior of the cells. Conversely, the larger AuNPs (100 nm) accumulate inside the cells because of the slow rate excretion; hence increase the mitochondrial toxicity^48^. Concerning the tissue distribution and nano-toxicity, Chen *et al*. reported that intra peritoneal injection of 5 nm AuNPs didn’t induce sickness or lethal effects in mice, pathological examination of different organs (liver, kidney lung, brain, heart, and spleen) didn’t show any abnormalities^49^. The authors added that 5 nm AuNPs were mainly excreted in urine.

Our findings indicated an overall improvement of DSS-induced adverse effects upon AuNPs treatment in DSS+ AuNPs-treated group. Body weight measurements significantly increased compared to the DSS group. DAI measurements were down regulated. Biochemical analyses revealed decreased MDA levels in colon homogenates, confirming the antioxidant effects of AuNPs. Similarly, AuNPs are able to inhibit lipids from peroxidation and prevent ROS formation, thus restoring the oxidative imbalances in acute inflammation model induced by carrageenan^50^. AuNPs are anti-oxidative agents that inhibit the generation of ROS and scavenge free radicals to improve antioxidant defence enzymes in diabetic mice^51^.

In the same group, the histopathological examination showed that the colon retained about normal structure except for some inflammatory cells. The low histological damage scores and morphometric analysis proved the previous findings. This might be attributed to the anti-oxidant and the anti-inflammatory effects of AuNPs. In agreement with the previous findings, other investigators reported that AuNPs reduced leukocyte migration following carrageenan injection^52^ and also macrophage infiltration in a model of arthritis^53^.

AuNPs interfere with transmission of inflammatory signaling. Sumbayev *et al*. debated that the anti-inflammatory activity of AuNPs was mostly attributed to extracellular interactions with IL-1β which aggregates around AuNPs, thus inhibiting IL-1β binding to cellular receptors^46^. AuNPs interact strongly with the cell membrane^54^ as well as also being internalized and trapped in endosomes^55^. Moreover, AuNPs ameliorated the inflammatory response by decreasing the mRNA expression of IL-1β, IL-6, TNF-α and inducible nitric oxide synthase^56^. Gold Nano-constructs are readily taken up by the reticulo-endothelial system^57^.

Interestingly, our study showed that AuNPs could ameliorate the progression of inflammation as evidenced by decreasing the pro-inflammatory cytokine IL-17 mRNA expression and immune-histochemical reactions in the colon compared to the DSS group. IL-17 coordinates tissue inflammation by inducing the expression of pro-inflammatory cytokines (such as IL-6 and TNFα and matrix metalloproteases, which mediate tissue infiltration and tissue destruction^58^. IL-17 is also a potent inducer for nitric oxide (NO) synthase and cyclooxygenase expression and this process is synergized by TNFα and IL-1β^59,60^. Consequently, IL-17 down regulation might partially contribute to reduce inflammation and counteract oxidative stress.

Analysis of Mallory’s trichrome-stained sections of the same group showed a highly significant decrease in collagen fibers when compared to DSS group. One of the anti-inflammatory mechanisms of action of gold compounds is inhibition of the synthesis of collagen in rheumatoid arthritis^61^. IL-17 exerts pro-fibrotic effects in the lung, liver, and heart. IL-17 deficiency reduces fibrosis in models of skin inflammation^62^, and treatment with IL-17 enhances cardiac fibroblast proliferation and migration in pulmonary fibrosis models^63^.

Accordingly, we postulated that IL-17 reduction might be partially implicated in the decrease of collagen fibers expression.

## Conclusion

Our study demonstrated a convenient, therapeutic effect of AuNPs that effectively targeted the colonic tissue, and reduced changes induced by DSS. The underlying mechanisms could be related to anti-oxidant and anti-inflammatory potential of AuNPs.

So, we recommend AuNPs as a novel therapeutic strategy in the treatment of UC. However, their administration to humans should be fully explored. Clinical trials with different doses and durations are needed to guarantee that therapeutic benefits will be balanced against any hazardous risks.

## Materials and Methods

### Chemicals

- Dextran sodium sulphate (DSS) [white powder; CAS No. 9011-18-1; M.W. = 40000 Da) was purchased from Alfa Aesar, Kandel, Germany
- Gold nanoparticles (AuNPs): (CAS No. 752568- naked) were purchased from Sigma–Aldrich (Steinheim, Germany) in the form of stabilized suspension in 0.1 mM phosphate buffered saline (PBS). They have the following properties: concentration ~5.5E + 13 particles/mL; core size 5 nm; optical density (OD) 1; hydrodynamic diameter 7–20 nm; formula weight 196.97; negative charge with a zeta potential in the range of −20 mV to −30 mV and storage temperature 2–8 °C.

### Experimental animals

Forty eight adult mice (BABL/c; 25–28 g weight; 8–12 weeks old) were obtained from the Breading Animal House, Faculty of Medicine, Zagazig University. They were housed in plastic cages with stainless steel wire-bar lid (2 mice per cage separated by partition) in a controlled room (temperature 18–23 °C, light cycle 14-h light/10-h dark, relative humidity of 40–60%) with food and water *ad-libitum*. Mice were acclimatized to this environment for two weeks before starting the experiment. All experimental procedures were performed in accordance with the guidelines of Institutional Animal Care and Use Committee and approved by Faculty of Medicine; Zagazig University (the protocol approval number: 3653). All data generated or analyzed during this study are included in this article.

### Experimental design

The mice were divided randomly into three main groups:

- Group I (control group): (24 mice) were equally subdivided into two subgroups (12 mice each); subgroup (Ia) was kept without treatment, subgroup (Ib) received AuNPs (2.5 mg/kg body weight/day) through intra-peritoneal injection starting on the 14^th^ day for 2 weeks^51^.
- Group II (DSS group): (12 mice) was administered 3% (w/v) DSS dissolved in drinking water. DSS was given in a cyclic manner to induce chronic colitis. Mice were placed on 3 cycles; each cycle consisted of 4 days of DSS followed by six days of drinking blank water^64^.
- Group III (DSS+ AuNPs group): (12 mice) was given DSS as DSS group and AuNPs (2.5 mg/kg body weight/day) through intra-peritoneal injection starting on the 14^th^ day for 2 weeks^51^.

To evaluate the optimal dose of AuNPs, we tried different concentrations. By real-time PCR analysis, we selected the dose of 2.5 mg/kg as the minimal therapeutic dose for accomplishing effective gene down-regulation of IL-17A (Supplementary Fig. 1) and to decrease the risk of adverse effects. Mass of AuNPs (g)/ml was 6.32 × 10^−5^ as provided by the supplier.

The selected dose was about 1/1000 of the LD50 of AuNPs (LD50: 2000 mg/kg body weight /day).

At the end of the experiment (after 1 month), mice were sacrificed by intra-peritoneal injection of sodium phenobarbital (50 mg/kg body weight)^65^. An incision through the abdominal wall was done to gain access to the colon which was carefully dissected (from cecum to rectum) and promptly washed with saline. The length of the colon of each animal was measured then the colon was divided into three parts, (i) the proximal third was stored at −80 °C to prepare tissue homogenates for biochemical analysis; (ii) the middle third was fixed in glutraldehyde for electron microscope examination; (iii) the distal third was fixed in 10% buffered formalin and processed for light microscope examination.

### Body weight measurements

During and at the end of the experiment, mice of all groups were weighed by digital electrical balance (Sartorius Goetting type 140/AG, W.Germany).

### Disease activity index (DAI) measurement

Severity of colitis was assessed by DAI based on the scoring system reported by Zong *et al*.^66^. During one month of treatment, the changes in body weight, visible stool consistency and rectal bleeding were recorded every 3 days. DAI is the summation of the weight loss index (0–4); stool consistency index (0–3); and faecal bleeding index (0–3). DAI = (combined score of weight loss, stool consistency and bleeding)/3. Scores were assessed as follows: for weight loss, a score of 0 for body weight within the 1% of baseline; 1 for a 1–5% loss; 2 for a 6–10% loss; 3 for an 11–15% loss and 4 for weight losses over 15%. For stool consistency, a value of 0 was assigned for well-formed pellets, 1 for pasty stools, and 3 for liquid stools. Rectal bleeding was graded 0 for none, 1 for occult bleeding, and 3 for gross bleeding. Occult blood in faeces was detected by using benzidine method (The principle of the test is based on that haemoglobin and its derivatives react in a similar way to peroxidase enzymes– by catalysing the oxidation of benzidine with production of a blue colour)^67^.

### Biochemical study

- Tissue MDA
  MDA was measured in colon homogenates by spectrophotometry according to the method of Ohkawa *et al*.^68^ using diagnostic kits (CAT. No. MD 25 29; Biodiagnostic Company, Dokki, Giza, Egypt). MDA was expressed as nmol/g protein.
- Real time - polymerase chain reaction (real time - PCR) for assessment of IL-17A expression

Total RNA was isolated from homogenates of mucosal extract of the colon using Qiagen RNA isolation kit (RNeasy, Qiagen Ltd, UK) following the manufacturer’s protocol. RNA was converted into cDNA by reverse transcriptase (QuantiTect Reverse Transcription Kit, Qiagen, Germany). The extracted RNA was used for the detection of expression of IL-17A genes. 18S rRNA was used as internal control. SYBR Green RT-PCR amplification was carried out using SYBR Green Real-time PCR Master Mix (Roche Diagnostics). The sequences of the primers used were: IL-17A forward, 5′-ATCCCTCAAAGCTCAGCGTGTC-3′ and reverse, 5′-ATCCCTCAAAGCTCAGCGTGTC-3′; 18S rRNA forward, 5′-CGGCTACCACATCCAAGGAA-3′ and reverse, 5′-GCTGGAATTACCGCGGCT-3′ ^69^. Amplification was followed by a melting curve analysis to check PCR product specificity. Results were normalized to 18S rRNA for stable expression (housekeeping gene). Relative changes of gene expression were calculated from the equation 2^−ΔΔCt^.

### Light microscope study

Specimens for light microscopy were fixed in 10% buffered formalin and processed to prepare 5-μm-thick paraffin sections for:

- H&E stain^70^
- Alcian blue stain^70^
- Mallory’s trichrome stain^70^
- Immunohistochemical stains for localization of intrleukin-17A (IL-17A) of by means the avidin-biotin complex (ABC) method (Thermo Scientific ABC Peroxidase Staining Kits, Code No. 32020, Rockford, USA) following the manufacturer’s instructions. Paraffin sections (5 μm), mounted on coated slides, were de-waxed and hydrated. To expose target proteins, antigen retrieval was performed using citrate buffer (pH6.0) microwaved for 15 minutes. Tissues were blocked in 3% bovine serum albumin for 30 minutes at room temperature. Then, they were incubated with the specific primary antibody overnight (4 °C): anti IL-17A (rat monoclonal antibody; Catalog # 11-7177-81; dilution 1/100; Thermo Fisher Scientific, WA, US). Endogenous peroxidase was quenched by incubation in 10% H_2_O_2_ in phosphate buffered saline (PBS) (pH 7.4). Detection was performed using biotinylated secondary antibodies and labeled horseradish peroxidase, followed by colorimetric detection by 3,3′-diaminobenzidine (DAB). Tissues were counterstained with Mayer’s hematoxylin and prepped for mounting. Omission of the primary antibodies served as negative controls. IL-17A positive cells showed brown cytoplasmic reactions^71^.

### Electron microscope study

Specimens for electron microscopy were immediately fixed in 2.5% phosphate-buffered glutaraldehyde (pH 7.4), post fixed in 1% osmium tetroxide in the same buffer at 4 °C and dehydrated. For transmission electron microscopy, the specimens were embedded in epoxy resin. Ultrathin sections (60–70 nm) were obtained (Leica ultra-cut UCT) and stained with uranyl acetate and lead citrate^72^, examined and photographed (JEOL JEM 2100 electron microscope; Jeol Ltd, Tokyo, Japan) in Electron Microscope Research Laboratory (EMRL) of Faculty of Agriculture, El Mansoura University, Egypt.

- Toluidine blue stain

Semi-thin sections (1 µm) were also obtained (Leica ultra-cut UCT), stained with toluidine blue and examined by light microscope.

### SEM and EDX analysis

SEM (JEOL JSM-6510LV electron microscope; Jeol Ltd, Tokyo, Japan) with X-ray analyzer used for EDX (X-Max^N^ 20 SDD system, Oxford Instruments, Oxford, UK) were used to detect the presence of AuNPs in the colonic tissue of DSS+ AuNPs group. In this procedure, the prepared sample was scanned to produce a high-resolution image for analysis. Then, EDX was used for determining elemental compositions.

### Morphometric study

Leica QWin 500 image analyzer computer system (Leica Ltd, Cambridge, UK) was used. The data was analyzed by Leica QWin 500 software with the aid of a digital camera connected to an optical microscope (Olympus, Tokyo, Japan) in the Image Analysis Unit at Pathology Department, Faculty of Dentistry, Cairo University. The measurements were performed by an investigator who was unaware of the experiment. The measuring frame of a standard area was equal to 7286, 78 µm^2^. Five non-overlapping fields were randomly chosen from each mouse in each group and examined to measure:

- Thickness of mucosa (µm) in H&E stained-sections at a final magnification (100x).
- Thickness of submucosa (µm) in H&E stained-sections at a final magnification (100x).
- Number of Goblet cells in alcian blue-stained sections at a final magnification (200x).
- Area percentage (%) of collagen fibers in Mallory’s trichrome-stained sections at a final magnification (200x).
- Area percentage (%) of positive IL-17A immunoreactions in anti-IL-17A immune-stained sections at a final magnification (200x).

H&E stained sections were scored to evaluate the histological damage using the scoring system described by Stillie and Stadnyk^73^. Slides were scored by a blinded histologist to grade the extent of inflammatory infiltrate (0–5), crypt damage (0–4), ulceration (0–3), and the presence or absence of edema (0 or 1). Histological damage score is the summation of the previous indices (maximum score = 13) (Table 4).

**Table 4:** Histological damage scoring system for quantitative evaluation of the colon injury.

Category	Score
**Inflammation**	
No infiltrate	0
Occasional, limited to submucosa	1
Significant Infiltrate in submucosa, limited to focal areas	2
Infiltrate in both submucosa and lamina propria, limited to focal areas	3
Abundant infiltrate in submucosa and lamina propria, covering large areas of mucosa	4
Transmural inflammation (mucosa to muscularis)	5
**Crypt damage**	
None	0
Some crypt damage, spaces between crypts	1
Loss of goblet cells, large spaces between crypts	2
Large areas without crypts, surrounded by normal crypts	3
No crypts	4
**Ulceration**	
None	0
Small, focal ulcers	1
Small, frequent ulcers	2
Large areas lacking surface epithelium	3
**Edema**	
Absent	0
Presen	1
**Histological damage score**	Sum (max = 13)

### Statistical analysis

Data for all groups were expressed as means ± standard error (X ± SE). Analysis was done using Statistical Package for Social Sciences (SPSS) version 22.0 (IBM Corp., Armonk, NY, USA). One-way analysis of variance (ANOVA) was used; followed by Tukey’s Honestly Significant Difference (Tukey’s HSD) test as a post hoc test for pairwise comparisons. The probability values (*P*) less than 0.05 were considered significant and highly significant when less than 0.001.

## Supplementary information

Supplementary figure 1

## Data Availability

All data generated or analysed during this study are included in this published article (and its Supplementary Information files).
