# Supplementary material for: Therapeutic effect of gold nanoparticles on DSS-induced ulcerative colitis in mice with reference to interleukin-17 expression
Source: Sci Rep. 2019 Jul 15;9:10176. doi: 10.1038/s41598-019-46671-1 (PMC6629650; doi:10.1038/s41598-019-46671-1)
Supplement: Supplementary file 1 — Supplementary figure 1 [file 41598_2019_46671_MOESM1_ESM.pdf]

Therapeutic effect of gold nanoparticles on DSS-induced ulcerative colitis in mice with reference to interleukin-17 expression

Amira M. Abdelmegid[1], Fadia K. Abdo[1], Fayza E.Ahmed[1], Asmaa A.A. Kattaia [1*]

[1] Department of Histology and Cell Biology, Faculty of Medicine, Zagazig University

[*] Corresponding Author

Tel.: +201220723273

E-mail addresses: asmaaalhosiny7@gmail.com; ehabmohomar@yahoo.com

Postal address: Faculty of Medicine, Zagazig University, Zagazig, Asharquia, Egypt. Postal code: 44519

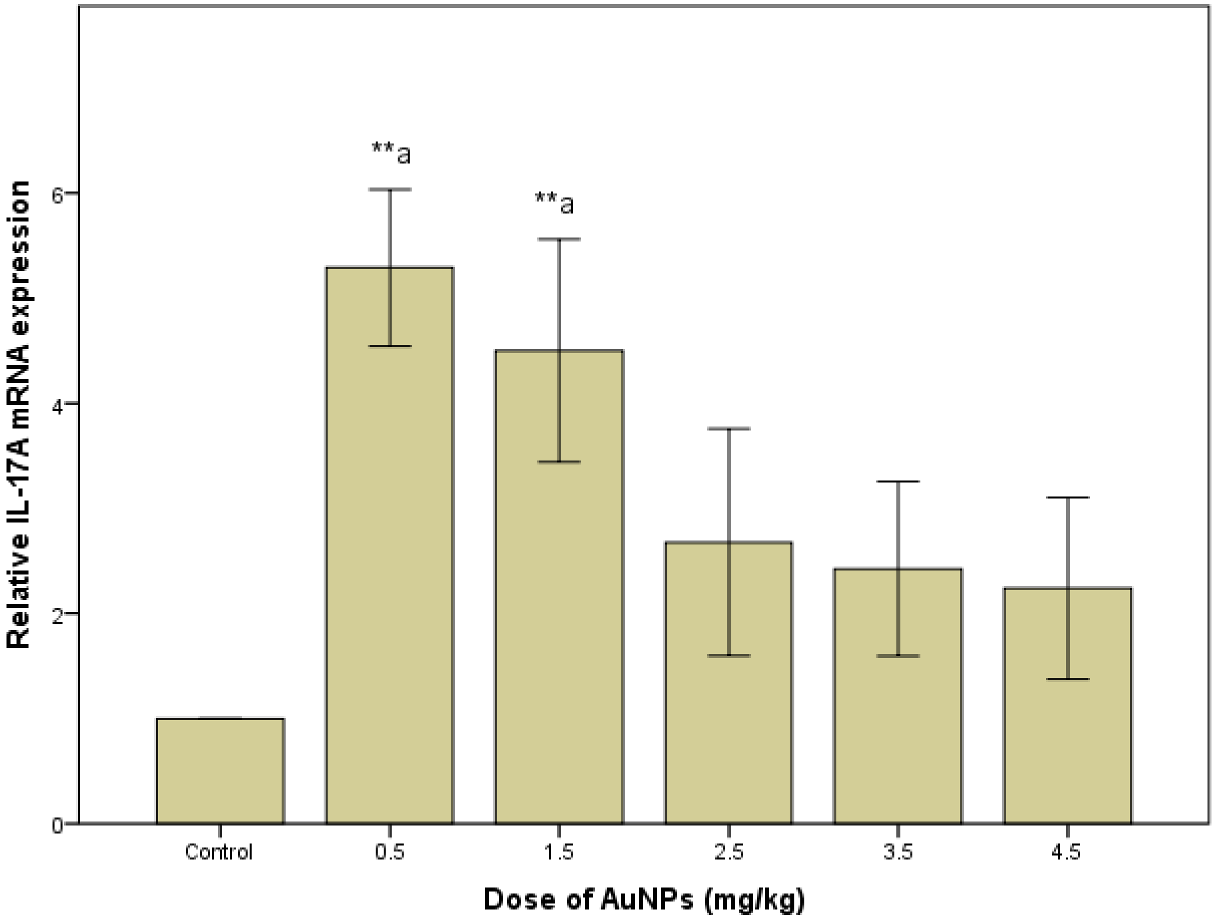

Supplementary figure 1. Real-time PCR analysis of IL-17A expression levels in the colons after administration of AuNPs at different concentrations. Values are estimated as a fold-increase compared to IL-17A expression in the calibrator control which is equal to 1. a: *P* compared with control group; Asterisks ** denote $p < 0.001$; n =6.
